# Modification of Layered Graphitic Carbon Nitride by Nitrogen Plasma for Improved Electrocatalytic Hydrogen Evolution

**DOI:** 10.3390/nano9040568

**Published:** 2019-04-08

**Authors:** Ming Gao, Danni Liu, Huanhuan Yang, Hao Huang, Qian Luo, Yifan Huang, Xue-Feng Yu, Paul K. Chu

**Affiliations:** 1 Shenzhen Institutes of Advanced Technology, Chinese Academy of Sciences, Shenzhen 518055, China; ming.gao@siat.ac.cn (M.G.); dn.liu@siat.ac.cn (D.L.); hh.yang@siat.ac.cn (H.Y.); hao.huang@siat.ac.cn (H.H.); qian.luo@siat.ac.cn (Q.L.); 2 University of Chinese Academy of Sciences, Beijing 100049, China; 3 Department of Physics and Department of Materials Science and Engineering, City University of Hong Kong Tat Chee Avenue, Kowloon 999077, Hong Kong, China; paul.chu@cityu.edu.hk

**Keywords:** layered graphitic carbon nitride, plasma treatment, electrocatalytic performance, hydrogen evolution reaction

## Abstract

As a layered nano-sheet material, layered graphitic carbon nitride (g-C_3_N_4_) has attracted attention in multifunctional photocatalytic applications. However, g-C_3_N_4_ is electrochemically inert consequently hampering electrochemical applications. In this work, low-temperature nitrogen plasma processing was conducted to modify g-C_3_N_4_ to enhance the electrocatalytic performance in the hydrogen evolution reaction (HER). The plasma produced significant morphological and chemical changes on the surface of g-C_3_N_4_ via active species, and nitrogen atoms were incorporated into the surface while the bulk properties did not change. The modification improved the surface hydrophilicity and electrocatalytic HER activity, as well as excellent stability in HER after 2000 cycles. Our results revealed that plasma treatment was a promising technique to improve the HER of carbon-based layered nano-sheet materials.

## 1. Introduction

Electrocatalytic water splitting employing electrocatalysts is a clean and efficient method to produce hydrogen as a sustainable and environmentally-friendly energy source [1]. In the hydrogen evolution reaction (HER), efficient and low-cost electrocatalysts are of great importance and noble metals such as platinum (Pt) are presently the most popular electrocatalysts [2]. However, the high cost and scarcity of noble metals have hindered broader application and non-noble metal HER catalysts composed of inexpensive and earth-abundant elements have been proposed [3]. Recently, with the discovery of graphene, layered nano-sheets materials have emerged as some of the most promising candidates for electrocatalysts because of their extraordinary physical and chemical properties [4]. Especially, as metal-free materials, carbon-based layered nano-sheet catalysts have aroused a great deal of interest by virtue of their tunable molecular structures, abundance, and robustness in acidic/alkaline environments [5].

As a metal-free polymeric semiconductor, layered graphitic carbon nitride (g-C_3_N_4_) has attracted attention in multifunctional catalytic applications such as photodegradation of pollutants, photocatalysis, CO_2_ reduction, and so on [6,7,8]. However, g-C_3_N_4_ is electrochemically inert, consequently hampering electrochemical applications. Much effort has been made to modify g-C_3_N_4_ to improve the electrocatalytic properties in HER. For instance, Zheng et al. have observed good HER activity from g-C_3_N_4_/nitrogen-doped graphene hybrids [9] and Tan et al. have demonstrated that several kinds of hybrid g-C_3_N_4_/p-doped graphene electrocatalysts have improved HER characteristics [10]. These strategies are based on external modification in conjunction of formation of hybrid composite and it is desirable to alter the intrinsic properties of g-C_3_N_4_. A recent study has shown that after sulfur doping, the HER activity of mesoporous carbon-supported g-C_3_N_4_ can be enhanced [11].

Plasma modification can be utilized to prepare high-performance catalysts by tailoring the surface properties or introducing dopants [12]. It has been reported that the abundant active species in the plasma can interact with the catalyst surface to change the physical and chemical properties, as well as the effects of plasma treatment on surface properties and electrocatalytic HER performance of graphene, molybdenum disulfide (MoS_2_), and cobalt phosphide (CoP) [13,14]. The argon plasma or oxygen plasma used in these experiments plays the main role as an etchant while some surface defects and vacancies are also created. In comparison, a nitrogen plasma can introduce N atoms or nitrogen-containing functional groups to the materials surface [15,16]. In this work, a low-temperature nitrogen plasma is employed to modify g-C_3_N_4_ to improve the electrocatalytic activity and nitrogen introduction alters the surface physical and chemical properties to enhance the HER performance while the favorable bulk attributes are preserved.

## 2. Materials and Methods

### 2.1. Materials Preparation

The layered g-C_3_N_4_ was synthesized from urea thermally. Briefly, 5 g urea (Analytical Grade, Sinopharm Chemical Reagent Co., Ltd.) were put into a covered crucible in a muffle furnace in air and heated to 550 °C for 2 h at a rate of 2.5 °C/min to obtain a yellow product. The nitrogen plasma treatment was carried out on a 40 kHz frequency inductively coupled discharge plasma system at a low pressure. The samples were put in the vacuum reactor and high-pure nitrogen (99.999%, Shenzhen Hongzhou Industrial Gases Co., Ltd., Shenzhen, China) was introduced into the reactor to maintain a pressure of 30 Pa. A power of 60 W was provided to form the discharge plasma and the plasma treatment was performed for different time durations at room temperature.

### 2.2. Materials Characterization

The morphology of the samples was observed on a Zeiss Supra 55 field-emission scanning electron microscope (SEM, Carl Zeiss, Oberkochen, Germany) and a FEI Tecnai G2 F30 transmission electron microscope (TEM, FEI Company, Oregon, OR, USA). The crystal structure of the catalysts was determined on a Rigaku MiniFlex 600 X-ray diffractometer (XRD, Rigaku Corporation, Tokyo, Japan) with Cu Kα radiation. The surface chemical composition was determined by X-ray photoelectron spectroscopy (XPS, Thermo Fisher ESCALAB 250 Xi, Waltham, MA, USA) using Mg Kα radiation and the binding energies were calibrated based on the standard C 1s peak (284.6 eV). The surface wettability was assessed by a water contact angle measurement based on the sessile drop technique on the Theta Lite instrument (Biolin Scientific, Gothenburg, Sweden). The measurements were repeated 5 times, and the averaged values and the standard deviation were calculated.

### 2.3. Catalytic Activity Evaluation

The electrocatalytic activity was assessed on a CHI 760E electrochemical analyzer (CH Instruments, Inc., Shanghai, China) using the standard three-electrode setup at ambient temperature with 0.5 M H_2_SO_4_ as the electrolyte. In the electrochemical tests, 1 mg of g-C_3_N_4_ was dispersed in 5 µL of 0.5 wt% Nafion and 995 µL of aqueous ethanol (1:1). The g-C_3_N_4_ modified glassy carbon electrode (GCE, diameter 3 mm) was prepared by casting 10 μL of the g-C_3_N_4_ suspension (1 mg·mL^−1^) on the GCE surface and was dried in air to form the working electrode. A graphite rod and saturated calomel electrode (SCE) were the counter electrode and reference electrode, respectively. The potentials were calibrated with respect to the reversible hydrogen electrode (RHE) as follows: E(RHE) = E(SCE) + (0.059 pH + 0.242) V. The onset overpotentials were determined based on the beginning of linear regime in the Tafel plot and iR compensation was applied to all the electrochemical measurements by impedance measurements. The cyclic voltammogram (CV) curves were recorded at a non-Faradic current from 0.190 to 0.306 V vs RHE at scanning rates of 5, 10, 20, 40, 80, 120, 160, and 200 mV·s^−1^. The double-layer capacitance was calculated as Cdl = j/r, where j was the current density and r was the scanning rate. The electrochemically active surface area (EASA) was calculated as: EASA = C_dl_/C_s_, where C_s_ was the specific capacitance value for a flat standard with 1 cm^2^ of real surface area and the average value was 60 μF·cm^−2^. The stability of the samples was tested by conducting CVs for 2000 cycles at a scanning rate of 100 mV/s.

## 3. Results and Discussion

### 3.1. Morphology and Crystalline Phases

**Figure 1:**
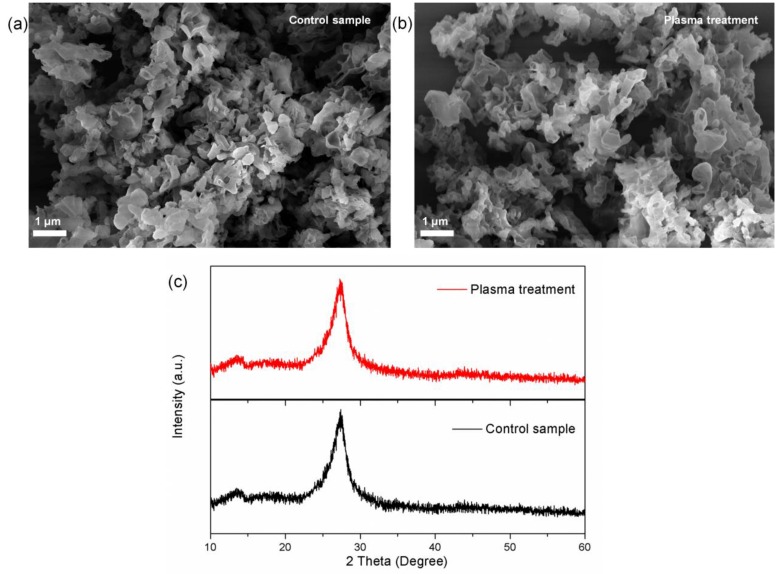
(**a**,**b**) SEM images, and (**c**) XRD patterns of the samples. (Plasma treatment time: 300 s; power: 60 W).

The surface morphology of the layered g-C_3_N_4_ before and after plasma treatment was observed by SEM and Figure 1a shows that the pristine sample was composed of solid micron-sized agglomerates. After treatment with the nitrogen plasma (Figure 1b), the agglomerates were preserved implying that high chemical stability of g-C_3_N_4_ and some g-C_3_N_4_ with a sheet-like morphology can also be observed. Plasma treatment modified the surface properties but hardly altered the bulk properties of materials. Figure 1c shows the XRD patterns of g-C_3_N_4_ before and after plasma treatment. The two diffraction peaks observed from both samples at 13.0°and 27.3° were the typical peaks of g-C_3_N_4_ (JCPDS card no. 87-1526). The stronger peak at 27.3° represented the (002) inter-planar stacking of the aromatic system and that at 13.0° corresponded to the (100) in-plane packing of the tri-s-triazine unit. These results confirmed that the molecular framework of pristine g-C_3_N_4_ was retained after the plasma treatment.

**Figure 2:**
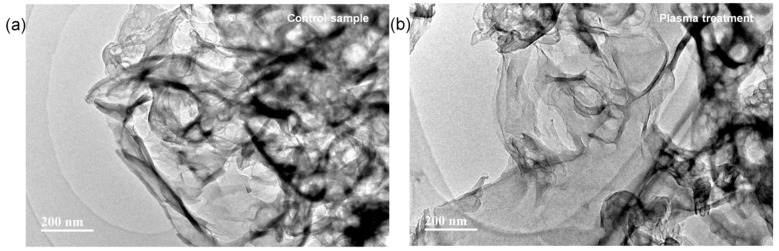
TEM images of (**a**) control, and (**b**) plasma-treated samples. (Plasma treatment time: 300 s; power: 60 W).

In plasma surface modification, etching effects is important [17] and the surface morphology was observed by TEM. As shown in Figure 2a, the control sample showed a densely stacked layered texture with an irregular shape. After the plasma treatment (Figure 2b), sheet-like nanostructures were seen indicating that active and energetic species in the discharge plasma converted the layered g-C_3_N_4_ into thin nanosheets, which was also found previously [18] and thin g-C_3_N_4_ nanosheets were observed to be efficient electrocatalysts compared to the bulk samples [19].

### 3.2. Surface Chemical Composition

**Figure 3:**
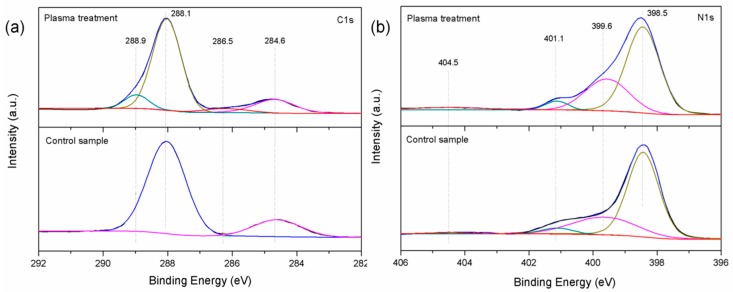
X-ray photoelectron (XPS) spectra of (**a**) C 1s, and (**b**) N 1s. (Plasma treatment time: 300 s; power: 60 W).

Besides changes in the surface morphology, the surface chemical composition was also altered by the plasma treatment. In comparison with the untreated sample (47.38% C1s, 52.13% N1s, and 0.48% O1s), the carbon content (41.72%) of the film surface modified by plasma decreased significantly while nitrogen (55.91%) and oxygen concentration (2.37%) increased. Figure 3a shows the C 1s XPS data revealing peaks at 288.1 eV and 284.6 eV from both samples corresponding to sp^2^ carbon in the aromatic ring (N–C=N) and C–C, respectively. The two new peaks at 286.5 eV and 288.9 eV after the plasma treatment can be ascribed to C–O/C–N and COOH [20,21]. This indicated that some of C–C or N–C=N bonds were broken and oxidized into the forms of COOH or C–OH groups. In the N 1s data (Figure 3b), three peaks can be deconvoluted, including that at 398.5 eV assigned to the sp^2^ hybridized nitrogen in triazine rings (pyridinic N), that at 399.6 eV related to the tertiary nitrogen of N–(C)_3_ groups (pyrrole N), and that at 401.1 eV corresponding to the primary amine groups. After plasma treatment, the intensity of pyrrole N increased suggesting that new three-coordinated nitrogen states were formed. This can be explained by the remediation of existing nitrogen vacancies through an ion-implantation process due to the nitrogen plasma [22]. The binding energy of the introduced N–C–O groups overlapped that of pyrrole N and it can also result in a corresponding increase [23]. The peak at 404.5 eV corresponding to N–N indicates that carbon was substituted by nitrogen [24]. The results showed that the nitrogen plasma treatment introduced a significant amount of nitrogen and created new functional groups on the g-C_3_N_4_ surface.

### 3.3. Surface Wettability

In electrocatalysis, good wettability with aqueous electrolytes is necessary [25]. The contact angle of the untreated sample was 78.3° and after the plasma treatment, it decreased to 55.2° indicating g-C_3_N_4_ became hydrophilic after plasma treatment. The change stemmed from nitrogen doping and the creation of polar functional groups on the surface by the molecular and atomic nitrogen plasma species [26].

### 3.4. Catalytic Activity and Stability

**Figure 4:**
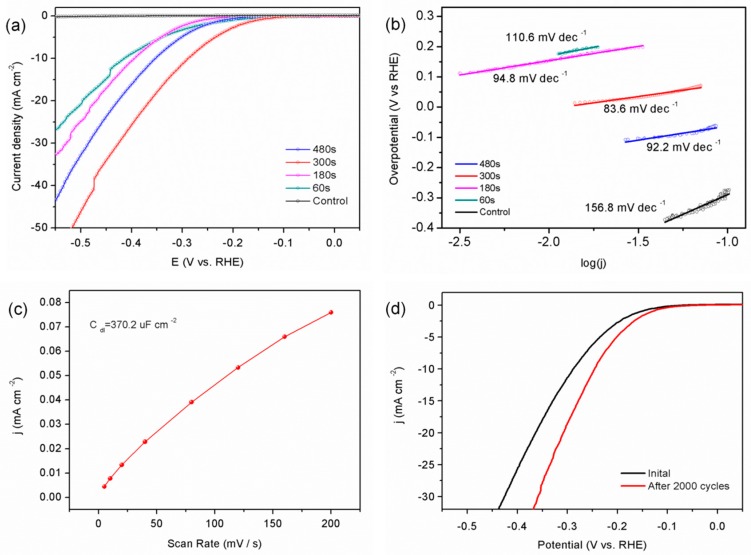
(**a**) Hydrogen evolution reaction (HER) polarization curves and (**b**) corresponding Tafel plots of the different catalysts; (**c**) double-layer capacitance (C_dl_) and (**d**) polarization curves before and after 2000 cycles of the N_2_ plasma 300 s sample.

Figure 4a shows the polarization curves acquired from g-C_3_N_4_ before and after the plasma treatment. The current density in HER of the pristine g-C_3_N_4_ was small and the polarization curve was almost a horizontal line versus the potential axis. On the other hand, the plasma-treated g-C_3_N_4_ delivered HER performance, reflected by a large shift of the polarization curve to a lower overpotential. The electrocatalytic HER activity was gradually enhanced with increasing treatment time from 60 s to 300 s. The sample treated for 300 s showed the best HER activity with a small onset potential of 288 mV to achieve a current density of 10 mA/cm^2^, which was much lower than that of the control one. However, if the plasma treatment time was further increased to 480 s, the HER performance worsened (10 mA/cm^2^ at 353 mV), possibly due to the excessive structural defects.

To further investigate the electrocatalytic activity of the treated g-C_3_N_4_, the linear portions of the Tafel plots were fitted to the Tafel equation. In principle, a relatively small Tafel slope indicates faster increment of the HER rate. As shown in Figure 4b, the Tafel slope of the pristine g-C_3_N_4_ was 156.8 mV·dec^−1^ and after the nitrogen plasma treatment, the Tafel slopes were 110.6 mV·dec^−1^, 94.8 mV·dec^−1^, 83.6 mV·dec^−1^, and 92.2 mV dec^−1^ corresponding to treatment time of 60 s, 180 s, 300 s, and 480 s, respectively. All of plasma-modified samples had smaller Tafel slopes than the control, indicating faster HER rates. The electrochemical double-layer capacitance (C_dl_), which can be used to determine the electrochemical active surface area (ECSA) was shown in Figure 4c. The N_2_-plasma 300 s catalyst exhibited a high capacitance of 370.2 μF·cm^−2^ and large ECSA for increased active sites. In a practical application, the electrocatalytic stability of the materials is a key parameter [27] and as shown in Figure 4d, the polarization curve of the plasma-treated sample after 2000 CV cycles showed an onset overpotential similar to that of the control one with negligible current loss, thus revealing excellent stability of the plasma-treated g-C_3_N_4_ under acidic conditions. This may because the physical structure and surface chemical composition after plasma treatment can be maintained. These results showed that nitrogen plasma treatment enhanced the HER activity and long-term stability of g-C_3_N_4_ significantly in an acid medium.

## 4. Conclusions

Low-temperature nitrogen plasma surface modification was performed to modify layered g-C_3_N_4_ to improve electrocatalytic performance. The plasma treatment changed the surface properties without affecting the bulk structure of g-C_3_N_4_. N_2_ plasma modification created thin g-C_3_N_4_ nanosheets and introduced new N and O functional groups on the g-C_3_N_4_ surface to enhance the HER activity. A hydrophilic surface, which enhanced contact with electrolytes, was also produced by the plasma treatment. Consequently, the plasma-treated sample has improved HER activity as well as excellent stability in HER after 2000 cycles. The results showed that plasma treatment was an excellent technique to improve the HER characteristics of carbon-based materials.
